# A Time-Resolved Diffusion Technique for Detection of the Conformational Changes and Molecular Assembly/Disassembly Processes of Biomolecules

**DOI:** 10.3389/fgene.2021.691010

**Published:** 2021-06-30

**Authors:** Yusuke Nakasone, Masahide Terazima

**Affiliations:** Department of Chemistry, Graduate School of Science, Kyoto University, Kyoto, Japan

**Keywords:** transient grating, time-resolved diffusion, reaction dynamics, protein-DNA interaction, stopped-flow

## Abstract

Biological liquid–liquid phase separation (LLPS) is driven by dynamic and multivalent interactions, which involves conformational changes and intermolecular assembly/disassembly processes of various biomolecules. To understand the molecular mechanisms of LLPS, kinetic measurements of the intra- and intermolecular reactions are essential. In this review, a time-resolved diffusion technique which has a potential to detect molecular events associated with LLPS is presented. This technique can detect changes in protein conformation and intermolecular interaction (oligomer formation, protein-DNA interaction, and protein-lipid interaction) in time domain, which are difficult to obtain by other methods. After the principle and methods for signal analyses are described in detail, studies on photoreactive molecules (intermolecular interaction between light sensor proteins and its target DNA) and a non-photoreactive molecule (binding and folding reaction of α-synuclein upon mixing with SDS micelle) are presented as typical examples of applications of this unique technique.

## Introduction

The dynamic and spatiotemporal compartmentalization of biomolecules is relevant for functional synchronization in living cells (Diekmann and Pereira-Leal, 2013). Biomolecular condensates due to the liquid–liquid phase separation (LLPS) are actively formed and dissolved in response to environmental stimuli for the compartmentalization (Brangwynne et al., 2009; Hyman et al., 2014; Banani et al., 2017; Shin and Brangwynne, 2017). The formation of LLPS is driven by dynamic and multivalent interactions among proteins and nucleic acids, and intrinsically disordered regions (IDRs) of proteins facilitate the assembly in many cases (Kato et al., 2012; Forman-Kay and Mittag, 2013; Nott et al., 2015). IDRs exist as a heterogeneous ensemble of conformations, which thermally fluctuates in solution (Oldfield and Dunker, 2014; Wright and Dyson, 2015). The flexibility would be a fundamental character to achieve the fluidity and reversibility of LLPS, since it provides a large interaction surface with a low binding affinity that are ideally suited for the transient and reversible interactions (Shammas, 2017; Tsafou et al., 2018). In the process of LLPS, target molecules are incorporated into the micro-droplet and the other molecules are excluded (Li et al., 2012; Lin et al., 2017). This molecular recognition system may also utilize the flexibility (Yang et al., 2019). During the assembly process, proteins interact dynamically with a lot of biomolecules, and upon binding to a target molecule, IDRs may form some specific conformations (e.g., cross-β structure) to stabilize the interaction (Kato et al., 2012; Xiang et al., 2015; Murray et al., 2017, 2018). This binding and folding processes should be key events for understanding the molecular mechanisms of LLPS. It has also been suggested that the IDRs maintain conformational heterogeneity in the condensed phase (Burke et al., 2015; Murthy et al., 2019). A variety of weak interactions such as hydrogen bonding, hydrophobic interaction and cation-π interaction via multiple residues contribute to stabilizing LLPS.

**FIGURE 1:**
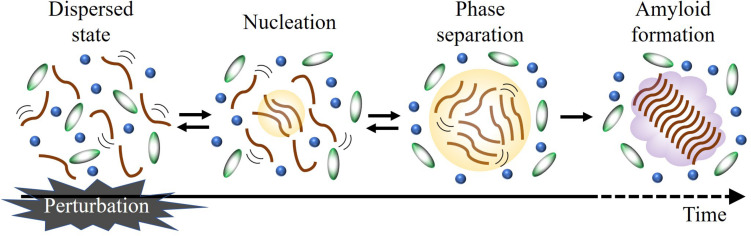
Schematic illustration of LLPS formation. Applying an external perturbation instantaneously, it is possible to detect the stepwise reaction in time-resolved manner.

Figure 1 shows a schematic diagram of the LLPS formation, which includes reversible transitions between dispersed and phase-separated states. In this scheme, a formation of seeds for LLPS (nucleation) is also illustrated. The nucleation may be a rather slow step which is followed by a rapid increase of the nucleus size, and eventually causes droplet formation (Tian et al., 2020). Recently, it has been reported that LLPS precedes the irreversible formation of amyloid hydrogels and fibrils (Wegmann et al., 2018; Ray et al., 2020; Xing et al., 2021). During this process, weak and transient interactions that govern the dynamic liquid phase are converted to more persistent interactions that stabilize the aggregate states. Since the rate constants at these steps determine the populations of these states and tendency of liquid-to-solid transition, LLPS and amyloid formation can be described kinetically if the rate constants are comprehensively determined. In order to understand a molecular recognition mechanism and the LLPS formation processes, it is also necessary to elucidate the intermolecular interaction of the transient state. For these purposes, it is required to investigate the LLPS process at the molecular level by time-resolved technique.

Liquid–liquid phase separation is controlled by various stimuli such as changes in ionic strength, pH, temperature, and post-translational modifications (Protter and Parker, 2016; Maharana et al., 2018; Ruff et al., 2018; Franzmann and Alberti, 2019; Gibson et al., 2019), which can be regarded as perturbations to the dynamic equilibria shown in Figure 1. If these perturbations are applied instantaneously (equilibrium jump), subsequent changes (relaxation processes) may be observed in a time-resolved manner to more deeply understand the properties of LLPS. To determine the rate constants and extract the structural information of the transient states, a time-resolved method which should have a high sensitivity to the conformation and oligomeric state of biomolecules is required.

A nuclear magnetic resonance (NMR) and Raman scattering spectroscopy have been used to study protein conformations in the dispersed, condensed, and aggregated states. Previous studies using these techniques have suggested that the IDRs do not form specific structures in the condensed phase, and liquid-to-solid state transition accompanies a loss of the flexibility by forming some specific structures (Burke et al., 2015; Murthy et al., 2019; Berkeley et al., 2021). Although these methods are useful to study LLPS on the molecular basis, the structural analyses are restricted to the equilibrium states due to their low time resolutions. Other methods such as small angle X-ray scattering (SAXS), atomic force microscopy (AFM), and fluorescence resonance energy transfer (FRET) have been used to investigate the conformational heterogeneities of IDRs (Mukhopadhyay, 2020; Surewicz and Babinchak, 2020; Bari and Prakashchand, 2021; Kodera et al., 2021). However, there are several difficulties to study the LLPS processes by these methods, although the time resolutions of them have been improved. For example, SAXS analyses would be less accurate in condensed phase due to interparticle interferences and a polydispersity of solution. AFM requires dilute solution for observation, making the analysis of LLPS difficult. Although the intra- and intermolecular interaction can be detected by monitoring the FRET efficiency, this measurement provides information on the length and angle between the two probe molecules in principle and determinations of the size and shape of the complex are difficult.

Here, we introduce a transient grating (TG) method as a unique spectroscopic technique that can detect the dynamic behavior of biomolecules in time domain. The TG method rapidly and precisely determines the diffusion coefficients of molecules (Terazima, 2006). The diffusion coefficient, *D*, is a physical property that represents the rate of molecular diffusion (Cussler, 2009), and is sensitive to the size and shape of a molecule and the intermolecular interaction with the solvent. For example, when association reaction occurs, *D* decreases due to an increase in a molecular size (Cussler, 2009). When the protein folds into a specific structure from a random coil, the hydrogen bonds with the solvent are switched to the intramolecular hydrogen bonds, which reduces the friction for the translational movement to increase *D* (Inoue et al., 2005). Therefore, the time-resolved detection of the *D*-change enables us to track the changes in the secondary, tertiary and quaternary structures of the protein. This technique is complementary to the other conventional methods and provides a comprehensive picture of conformational changes and intermolecular assembly/disassembly reactions in time domain.

In this review, we firstly describe the principle of the TG method and how to analyze the diffusion signal. Secondly, as application examples of the TG method, recent studies on protein–protein and protein-DNA interactions of photoreactive proteins are presented. Thirdly, the protein-micelle interaction of a non-photoreactive protein studied by the TG method with a stopped flow apparatus is presented. Observation of such dynamics should be helpful to understand the molecular mechanisms of enzymatic reactions, signalings, and LLPS formation processes.

## Transient Grating Method

**FIGURE 2:**
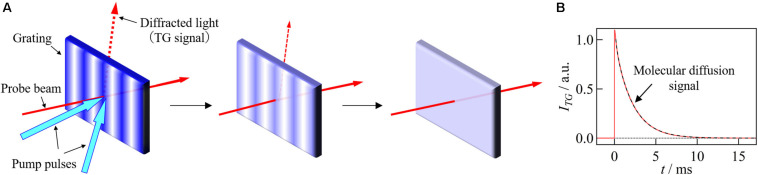
**(A)** A schematic drawing of the TG method. The grating created by the interference pattern of the excitation light disappears with the diffusion process, which is detected as a decay of diffracted light intensity (TG signal). **(B)** TG signal representing the molecular diffusion obtained for the β-lactoglobulin labeled by a photochromic compound (spiropyran) is shown. Solid red line represents the TG signal and broken black line represents the fitting curve based on Eq. 2 with *D*_R_ = *D*_P_.

The TG method is one of four-wave mixing spectroscopies and has been used for detection of weak absorption or studying excited state dynamics in physics. From the perspective of elucidating chemical reactions, this technique was used for studying translational diffusion processes of short-lived species such as transient radicals for the first time (Terazima and Hirota, 1993), and anomalously slow diffusions of the radicals have been discovered (Terazima, 2000). Later, some methods based on the TG technique have been developed to measure the reaction volumes and reaction enthalpies (Terazima et al., 1995), and these methods have been applied to studies on reaction mechanisms of proteins (Takeshita et al., 2000; Sakakura et al., 2001). In the TG method, two pulsed laser beams are crossed into the sample solution and the target molecules are photoexcited by an optical interference pattern created by the two beams (Figure 2A; Eichler et al., 1986; Terazima, 2002). For example, a chromophore of a light sensor protein is excited by the interference pattern, which triggers photoreaction of the target protein. Due to a variety of physical and chemical processes after the photoexcitation, sinusoidal modulations in the refractive index (*n*) and the absorption (κ) are created. This spatial modulation works as a grating, which is monitored by the diffraction of a continuous wave probe beam. The probe beam is crossed at the grating region with an appropriate angle to satisfy the phase-matching condition (Bragg condition) (Eichler et al., 1986). The diffracted light is called TG signal. In the case of weak diffraction, the grating intensity [I_TG_(*t*)] is approximately proportional to the squares of the amplitudes of the modulations in the refractive index (δ*n*) and absorption changes (δ*κ*):

(1) $$I_{\text{TG}}\left(t\right)≅\alpha {\left(\delta n\right)}^{2}+\beta {\left(\delta \kappa \right)}^{2}$$

where α and β are constants that represent the sensitivity of the system. In the TG method, the wavelength of the probe light does not have to be in the absorption band of the target molecule. In this case, only the contribution of the refractive index change appears as the signal. Since the refractive index change is caused by the heat release due to the non-radiative deactivation of the excited state, changes of absorption spectrum, and a partial molar volume change due to a chemical reaction, these properties can be obtained quantitatively in addition to the information obtained by absorption measurement (Terazima, 2002, 2004; Iwata et al., 2018).

After fast photochemical reactions, the concentration modulations of the reactant and the photo-product gradually disappear due to the translational diffusion. The intensity of the TG signal decays by this process, and the decay rate depends on the molecular diffusion rate (Figures 2A,B). An important point is that the grating length is very short (on the order of μm), hence, *D* can be measured in a short time (∼ms). Conventional techniques, such as the Taylor dispersion, dynamic light scattering, fluorescence correlation spectroscopy, and NMR require several minutes to several days for the measurements (Alizadeh et al., 1980; Price, 1997; Elson, 2011; Stetefeld et al., 2016), and are limited to the observation of relatively stable species. The TG method, on the other hand, can determine *D* for short-lived species, enabling the detection of biomolecular reactions (conformational change of protein part, intermolecular assembly and disassembly) from a viewpoint of the *D*-change (Terazima, 2006, 2011).

If all chemical reactions are completed before the observation time of the diffusion signal, the TG signal may be expressed by

(2) $$I_{\text{TG}}\left(t\right)=\alpha {\left\{\delta n_{\text{p}}\text{exp}\left(-D_{\text{P}}q^{2}t\right)-\delta n_{R}\text{exp}\left(-D_{\text{R}}q^{2}t\right)\right\}}^{2}$$

where the subscripts P and R stand for the product and the reactant, respectively, and δn_P_(_R_) is the peak-to-null refractive index difference in the sinusoidal modulation at *t* = 0. The negative sign for δn_R_ comes from the 180° phase difference of the fringe pattern of the reactant from that of the product due to the consumption of the reactant. The *q* is a grating wavenumber, which can be controlled by changing the crossing angle of the two excitation pulses. The larger the grating wavenumber is, the faster the diffusion signal appears, since diffusion over short distance homogenizes the solution quickly. The *q*^2^ value can be determined from the decay rate of the thermal grating signal of a calorimetric reference sample, which releases all photon energy as a heat, and the thermal diffusivity (*D*_th_) under the experimental conditions.

**FIGURE 3:**
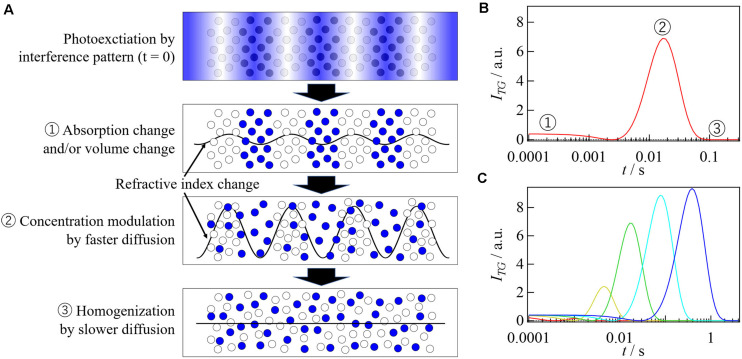
**(A)** The molecular diffusion processes and changes in the refractive index modulation during the TG measurement are illustrated. **(B)** Simulated TG signal for the reaction which accompanies the *D*-change upon photoexcitation. The numbers indicated in this figure correspond to the states illustrated in **(A)**. **(C)** Simulated TG signals obtained under several *q*^2^ condition for the reaction represented by the Scheme 1. *D* values are 8.0 × 10^– 11^, 7.8 × 10^– 11^, and 6.0 × 10^– 11^ m^2^/s for reactant, intermediate, and product, respectively, and the rate constant of the *D*-change (*k* in Scheme 1) is 500 s^– 1^. The *q*^2^ values are 3000, 500, 100, 20, 4.0 × 10^10^ m^– 2^ from left to right.

The shape and intensity of the molecular diffusion signal strongly depend on *D* of the reactant and product. When *D* does not change upon photoexcitation, the two terms in Eq. 2 cancel each other and a single exponential decay signal appears (Figure 2B). If *D* changes upon photoexcitation, the amplitude of the refractive index modulation may transiently increase due to the fast diffusion of the chemical species having a larger *D*, as shown in Figure 3A. This concentration modulation finally disappears due to the homogenization of the solution. Therefore, the TG signal shows a rise-decay profile as shown in Figure 3B, and the peak intensity increases as the difference between *D*_R_ and *D*_P_ increases.

As long as both *D*_P_ and *D*_R_ are time independent in the observation time window, the diffusion signal can be described by Eq. 2 and the peak intensity should not depend on *q*^2^. If *D* changes in the time scale of the diffusion signal, however, the intensity and shape of the diffusion signal depend on *q*^2^ significantly (Figure 3C). In such a case, the observed diffusion signal should be analyzed using a proper model. As an example, a typical reaction scheme is shown below.

$$\text{Scheme 1}\;R\overset{h\nu }{⟶}I\overset{k}{⟶}P$$

where R, I, P, and *k* represent the reactant, the intermediate, the final product and the rate constant of the change, respectively. Solving the reaction-diffusion equation based on this model, δ*n*_R_(*t*) and δ*n*_P_(*t*) are given by

(3) $$\begin{array}{c} \delta n_{R}\left(t\right)=\delta n_{R}\exp\left(-D_{R}q^{2}t\right)\\ \delta n_{P}\left(t\right)=\delta n_{I}\text{exp}\left\{-\left(D_{I}q^{2}+k\right)t\right\}+\delta n_{P}\frac{k}{\left(D_{P}-D_{I}\right)q^{2}-k}\\ \left[\text{exp}\left\{-\left(D_{I}q^{2}+k\right)t\right\}-\text{exp}\left(-D_{P}q^{2}t\right)\right] \end{array}$$

where δ*n*_I_ and *D*_I_ are the initial refractive index change due to the creation of the intermediate species and *D* of intermediate species, respectively. By global analyses of the diffusion signals obtained at various *q*^2^ using Eq. 3, *D* of each species and the rate constant of the *D*-change can be determined. Similarly, theoretical equations for more complicated reactions can be derived.

For demonstrating the potential of the TG method and for showing recent extension of the TG method, studies on photoreactive molecule (intermolecular interaction between light sensor proteins and its target DNA) and non-photoreactive molecule [folding reaction of α-Synuclein (αSyn) upon binding of SDS micelle] are presented in this review.

## Studies on Photoreactive Proteins

### Protein–Protein and Protein-DNA Interaction Dynamics

Protein-DNA interaction is a fundamental and ubiquitous process for a variety of biological functions. Consequently, it has been studied using various methods such as electrophoretic mobility shift assay, DNA pull down assay, and surface plasmon resonance (Campbell and Kim, 2007; Dey et al., 2012; Jutras et al., 2012; Chaparian and van Kessel, 2020). However, kinetic information has been limited, because of a lack of experimental method to initiate and detect the intermolecular interaction dynamics. In these respects, photosensory proteins are useful because their reactions can be triggered by light and TG method is appropriate to detect the intermolecular interaction.

**FIGURE 4:**
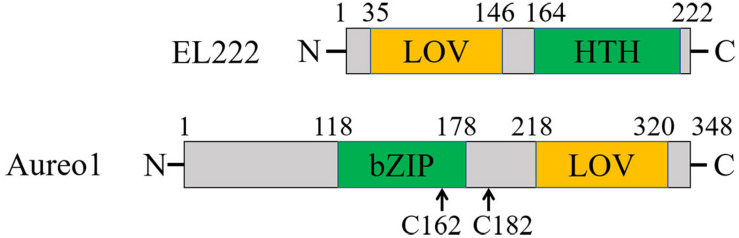
Domain structures of EL222 and Aureo1. The two cysteine residues replaced by serines to prevent the formation of disulfide bonds are indicated for Aureo1.

EL222 from a bacterium *Erythrobacter litoralis* HTCC2594 and Aureochrome-1 (Aureo1) from a green alga *Vaucheria frigida* are blue light sensor proteins, both of which have a light-oxygen-voltage (LOV) domain as a photoreceptor domain (Figure 4; Takahashi et al., 2007; Nash et al., 2011; Rivera-Cancel et al., 2012; Takahashi, 2016). They regulate the DNA transcription in a light-dependent manner to control several biological responses such as a photomorphogenesis in *V. frigida*. The LOV domain non-covalently binds a flavin mononucleotide (FMN) as a chromophore (Crosson and Moffat, 2001). Upon light illumination, it forms a covalent adduct with a nearby cysteine residue (Conrad et al., 2014), which triggers conformational changes in the protein, and the intermolecular interaction changes to transmit the light signal for functioning (Crosson and Moffat, 2001; Harper et al., 2003; Herrou and Crosson, 2011; Terazima, 2011). EL222 has a helix-turn-helix (HTH) domain and Aureo1 has a bZIP domain as their DNA-binding domains (Figure 4; Takahashi et al., 2007; Nash et al., 2011). Under conditions that inhibit intermolecular disulfide bonds, both proteins exist as the monomers in the dark, and have low DNA-binding abilities (Rivera-Cancel et al., 2012; Hisatomi et al., 2014). Upon light irradiation, they both bind to DNA as the dimeric forms. However, the intermolecular interaction dynamics and the recognition mechanism of the target sequence had remained unclear. Hence, the photoreaction dynamics of EL222 and Aureo1 in the absence and presence of DNA were studied by the TG method (Akiyama et al., 2016; Takakado et al., 2017, 2018).

### Reaction Dynamics of EL222 and Its Interaction With DNA

**FIGURE 5:**
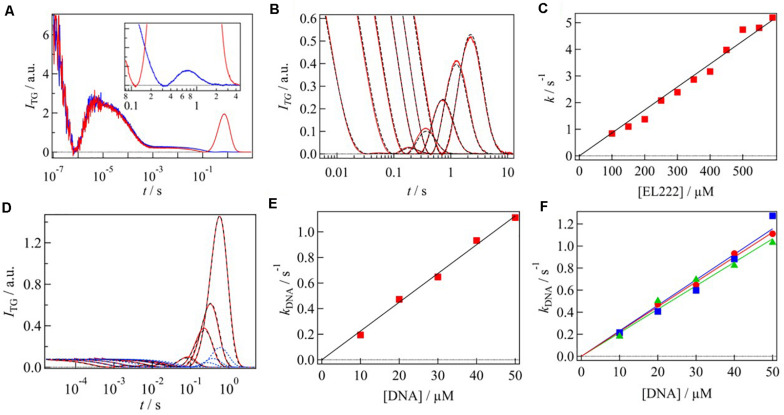
**(A)** TG signals of EL222 in the absence (blue) and presence (red) of DNA. The molecular diffusion signal of EL222 without DNA is enlarged in the inset. **(B)** *q*^2^ dependence of diffusion signal of EL222 in the absence of DNA. The signals were normalized by the number of photoexcited molecule. The fitting curves based on Eq. 3 are shown in black broken lines. **(C)** Rate constant of *D-*change of EL222 was plotted against the EL222 concentration. **(D)** *q*^2^ dependence of diffusion signal of EL222 in the presence of DNA (red lines). The fitting curves based on Scheme 2 and Scheme 3 are shown in blue and black broken lines, respectively. **(E)** Rate constant of *D-*change associated with the DNA binding was plotted against the DNA concentration. **(F)** Rate constants of DNA bindings for the three different sequences were plotted against the DNA concentration. Red, blue, and green plots correspond to sequences 1–3, respectively. All figures are adapted from Takakado et al. (2017, 2018) with slight modifications.

Typical TG signals of EL222 after photoexcitation at 450 nm in the absence and presence of target DNA are shown in Figure 5A. The signals on the fast time scale (100 ns to 1 ms) were almost identical. The signals rose quickly followed by decay–rise and decay components. These phases were assigned to the adduct formation between the chromophore and the cysteine residue with a time constant of 0.7 μs, and the thermal diffusion. A significant difference between the two signals was observed in the slower time region, that is, the intensities of the rise-decay signals are quite different.

Since the time scale of the rise–decay signal depended on *q*^2^, it was attributed to the diffusion process. The rise-decay profile clearly indicates that *D* is changed by the photoreaction. Based on the signs of the refractive index changes of these phases, it was found that the rise and decay components represent the diffusion of the reactant and the product, respectively, indicating slower diffusion of the product compared with that of the reactant (*D*_R_ > *D*_P_). By addition of DNA to the solution, the diffusion signal intensity increased, and the decay rate of the diffusion signal slowed down, indicating the decrease in *D*_P_ upon the addition of DNA. The peak intensity of the diffusion signal depends on the difference between *D*_P_ and *D*_R_. In this case, since DNA is not photoexcited by the excitation pulse used here, the reactant which creates the grating is a monomer of EL222 and *D*_R_ should not be changed by the addition of DNA. Hence, the enhancement of the diffusion peak indicated also the decrease in *D*_P_. The smaller *D*_P_ was attributed to the interaction of the photoexcited EL222 with the DNA.

The protein–protein and protein-DNA interaction dynamics were determined by analyzing the *q*^2^-dependence of the molecular diffusion signal. Figure 5B shows the diffusion signals of EL222 in the absence of DNA at various *q*^2^. The signals were normalized by the number of the photoexcited molecules. The intensity of diffusion signal was stronger at a slower time, showing that the *D*-change occurs in this time window. The observed TG signals were analyzed based on Scheme 1. The signals were reproduced well and the rate constant of the *D*-change (*k*) was determined to be 3.9 s^–1^ at a protein concentration of 350 μM. The parameters, such as *D*, obtained by the analyses are listed in Table 1.

**TABLE 1:** Kinetic parameters and diffusion coefficients obtained by the analyses of TG signals of EL222 and AureoCS are listed (Akiyama et al., 2016; Takakado et al., 2017, 2018).

	Reaction rates				Diffusion coefficients	
	Adduct form	Dimerization	DNA binding	DNA dissociation	Reactant	Final product
EL222	1.4 × 10^6^ s^–1^	8.6 × 10^3^ M^–1^ s^–1^	ND	ND	8.8 × 10^–11^ m^2^ s^–1^	6.6 × 10^–11^ m^2^ s^–1^
EL222 + DNA	1.4 × 10^6^ s^–1^	9.3 × 10^3^ M^–1^ s^–1^	2.2 × 10^4^ M^–1^ s^–1^	ND	8.8 × 10^–11^ m^2^ s^–1^	5.7 × 10^–11^ m^2^ s^–1^
AureoCS	4.8 × 10^6^ s^–1^	2.8 × 10^3^ M^–1^ s^–1^	ND	ND	8.1 × 10^–11^ m^2^ s^–1^	5.3 × 10^–11^ m^2^ s^–1^
AureoCS + DNA	4.8 × 10^6^ s^–1^	2.8 × 10^3^ M^–1^ s^–1^	7.7 × 10^4^ M^–1^ s^–1^	0.26 s^–1^	8.1 × 10^–11^ m^2^ s^–1^	3.7 × 10^–11^ m^2^ s^–1^

*ND, not determined.*

To clarify the origin of the *D*-change, the protein concentration dependence on the diffusion signal was examined. The shape and intensity of the diffusion signal strongly depended on the protein concentration (Takakado et al., 2017). The higher the concentration was, the stronger the signal was. This dependence was explained by a faster rate of the *D*-change (*k*) at higher concentrations; i.e., the *D*-change comes from a multimolecular reaction. Since the apparent rate constant of the *D-*change (*k*) linearly depended on the concentration of EL222 (Figure 5C), it was concluded that the reaction was a bimolecular reaction, that is, the dimerization. From the slope of the plot, the second order rate constant of the protein dimerization was determined to be 8.6 × 10^3^ M^–1^ s^–1^.

Next, to determine the intermolecular interaction dynamics with DNA, the TG signals were obtained at various *q*^2^ in the presence of the target DNA (Figure 5D). It showed significant time development, representing that the *D*-change (intermolecular interaction with DNA) occurs in the observation time window. There may be two possible reaction schemes for producing the final product EL222(dimer)-DNA complex: dimerization occurs before DNA binding (Scheme 2), and DNA binding occurs before dimerization (Scheme 3).

$$\text{Scheme 2}\;R\overset{h\nu }{⟶}I\overset{k_{1}}{⟶}\text{dimer}\overset{k_{2}}{⟶}\text{dimer-DNA}$$

$$\text{Scheme 3}\;R\overset{h\nu }{⟶}I\overset{k_{1}}{⟶}\text{monomer-DNA}\overset{k_{2}}{⟶}\text{dimer-DNA}$$

The time-profiles of the diffusion signals based on these two schemes are different, and the TG signals were well reproduced not by Scheme 2 but by Scheme 3 (Figure 5D). This represents that EL222 binds DNA as a monomeric form, which is followed by the dimerization.

The second-order rate constant of the DNA binding (*k*_1_ in Scheme 3) was determined by the TG signals at various concentrations of DNA with a constant protein concentration. The shape and intensity of the diffusion signal depended on the DNA concentration (Takakado et al., 2018). These signals were reproduced by Scheme 3 and the apparent rate constants of the DNA binding process were plotted against the concentration of DNA (Figure 5E). From the slope of the plot, the second-order rate constant was determined to be 2.2 × 10^4^ M^–1^ s^–1^, which was faster than that of protein dimerization.

A detailed mechanism of the DNA binding was studied by the DNA sequence dependence of the DNA binding rate. Three sequences of the DNA fragment with the same length (45 bp) were used (Takakado et al., 2018); sequence 1: 5′-GGTAGGATCC ATCGGGCAGTGCGGTCAGCGGCATGCCGGCAG-CAG-3′, sequence 2: 5′-TTGCGAGAAGAAAATATGGACCTTGGCCCA TGATGGACACAAT-AC-3′, sequence 3: 5′-AAAAAAAAAA AAAAAAAAAAAAAAAAAAAAAAAAAAAAAAAA-AAA-3′. They have different binding affinity to EL222 and the concentrations of EL222 at half-maximum binding (EC_50_) were 13 μM (sequence 1), 2.4 μM (sequence 2), and 18 μM (sequence 3). By the same analyses described above (Figure 5F), the second-order rate constants of the DNA binding were determined as 2.2 × 10^4^ M^–1^ s^–1^ for sequence 1, 2.3 × 10^4^ M^–1^ s^–1^ for sequence 2, and 2.1 × 10^4^ M^–1^ s^–1^ for sequence 3. The reaction rate constants of the DNA binding were almost identical, despite their different binding affinities. This indicates that the dissociation rate depends on the sequence, but the association reaction occurs similarly. This observation must be relevant to the molecular recognition.

### Reaction Dynamics of Aureo1 and Its Interaction With DNA

The photoreaction of Aureo1 and its interaction with DNA were also studied by the TG method. A construct consisting of the functional bZIP and LOV domains (113–348 aa) forms a dimer by intermolecular disulfide linkages at Cys162 and Cys182 (Hisatomi et al., 2013). By replacing the cysteines to serines (AureoCS), the dimer dissociates into the monomer in the dark. This monomer construct undergoes the dimerization upon photoexcitation, and the affinity with the target DNA increases (Hisatomi et al., 2014). Since the oxidation-redox potentials of the two Cys residues are close to that in the cell (Hisatomi et al., 2014), monomeric Aureo1 is proposed to present in a living cell, and the light-induced dimerization may be coupled with the change in affinity for DNA. The photoreaction of AureoCS and its interaction with DNA (5′-GACCTGAG TGCTCGAGCTGCGAGACGCTGTC-TGACGTCAGACAGCG TCTCGCAGCTCGAGCACTCAGGTC-3′) was studied by the TG method to determine the reaction scheme and kinetics (Akiyama et al., 2016).

**FIGURE 6:**
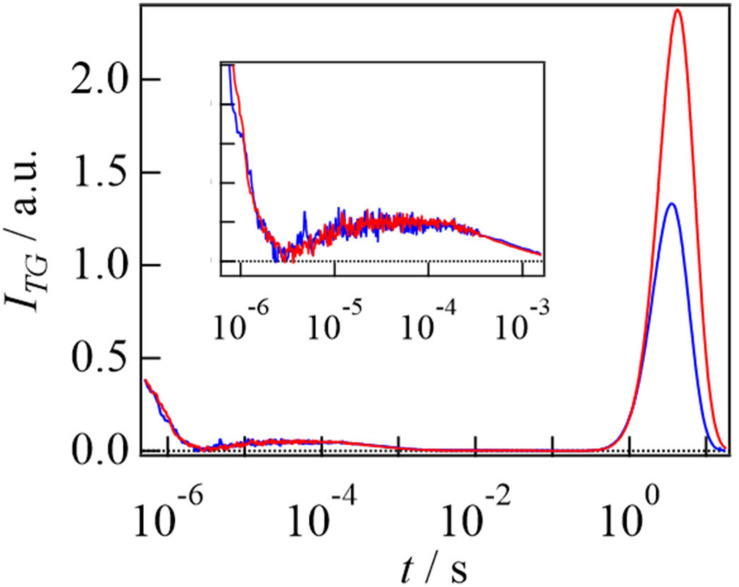
TG signals after photoexcitation of AureoCS (20 μM) (blue line) and of AureoCS (20 μM) with the target DNA (10 μM) (red line). The TG signals on the short time range are enlarged in the inset. The TG signals are adapted from Akiyama et al. (2016) with minor modifications.

Figure 6 shows the TG signals of AureoCS with and without DNA. As the case of EL222, the TG signal of AureoCS with DNA was identical to that of AureoCS without DNA in a short time range. The temporal profiles are also similar to that of EL222. This similarity is reasonable, because both proteins have the LOV domains as the light sensing modules and their photochemistries are well conserved (adduct formation between the FMN chromophore and nearby Cys residue) (Conrad et al., 2014). The time constant of the adduct formation was determined to be 2.1 μs.

The strong rise-decay component observed in a slower time region was assigned to the molecular diffusion signal. From the signs of the refractive index changes, the rise and decay components were respectively attributed to the diffusion processes of the reactant and product (*D*_R_ > *D*_P_). By adding DNA at a small *q*^2^, the intensity of the diffusion peak was enhanced and the signal was shifted to a slower time scale. These behaviors are similar to the EL222 reaction, and these changes imply that photoexcited AureoCS interacts with DNA.

Based on the *q*^2^ dependence and concentration dependence of the diffusion signal in the absence of DNA, it was clarified that AureoCS undergoes dimerization and its second order rate constant was determined to be 2.8 × 10^4^ M^–1^ s^–1^. Next, the photoinduced intermolecular interaction change with the target DNA was studied. If the photoexcited monomeric AureoCS binds the DNA before the dimerization reaction, a *D*-change should be detected as a rise-decay diffusion signal on a short time scale. However, the TG signal did not show any *D*-change before the dimerization (Akiyama et al., 2016). Hence, it was concluded that the photoexcited monomeric AureoCS does not bind the target DNA. On the other hand, the target DNA influences the TG signal in a longer time range (Figure 6), in which the photoexcited AureoCS dimerized, indicating that the target DNA interacts with the photo-dimerized AureoCS.

The kinetics of the protein-DNA interaction was elucidated by the *q*^2^ dependence of the diffusion signal. Based on the model that the DNA binding occurs after the dimerization, the signals were reproduced well, and the rate constant of the DNA binding was determined to be 0.98 s^–1^ at a DNA concentration of 10 μM. The DNA binding rate constant is slightly larger than that of the dimerization of AureoCS at the concentration of 20 mM (0.52 s^–1^). Hence, the dimerization should be the rate-determining step for the DNA binding. From the analyses of the DNA concentration dependence of the TG signal, the second order reaction rate of the DNA binding and its dissociation rate were determined to be 7.7 × 10^4^ M^–1^ s^–1^ and 0.26 s^–1^, respectively. From these data, the dissociation constant of the DNA binding in the light state (*K*_d_) was determined to be 3.4 μM. It is interesting that the second-order rate constant of the DNA binding (7.7 × 10^4^ M^–1^ s^–1^) is about three-times larger than that of the dimerization of AureoCS (2.8 × 10^4^ M^–1^ s^–1^). Therefore, the rate-determining step must be the protein dimerization, and the recognition of the target DNA is faster.

**FIGURE 7:**
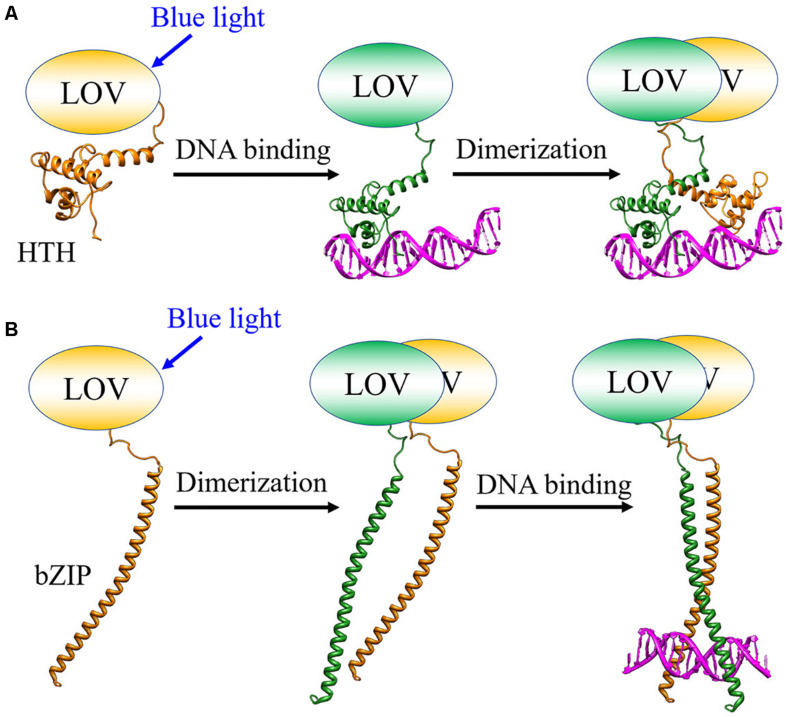
Photoinduced intermolecular interaction dynamics of **(A)** EL222 and **(B)** Aureo1 studied by the TG method.

As described above, the light induced protein–protein and protein-DNA association reactions were successfully detected. Kinetic analyses revealed that EL222 binds to DNA in the monomeric state before the dimerization, while Aureo1 binds to DNA after the dimerization (Figure 7). Although the reactions of Aureo1 and EL222 are very similar as far as the absorption changes (chromophore reactions) are monitored, the TG measurements revealed the differences in the signal transduction mechanism and the dynamics of association with DNA. These findings can be obtained only by the time-resolved measurements, and the similar analyses will be applied to the formation process of LLPS.

Since the LLPS formation is often triggered by the presence of RNA and DNA (Langdon, 2018; Shakya and King, 2018; Turner et al., 2018; Zhou et al., 2019; Roden and Gladfelter, 2021), nucleic acids can have a significant effect on LLPS due to their physical and chemical properties. On this point, it may be interesting to note that the reaction yield of the dimerization of EL222 was enhanced by the presence of DNA (Takakado et al., 2017, 2018). This suggests that the initial DNA binding step enhances the protein dimerization by changing the protein structure. We speculate that the LLPS formation may be triggered by similar mechanisms, that is, the IDRs form some specific structure upon binding DNA which leads to further assembly of molecules.

Recently, by genetically combining a photosensor protein with a protein which mainly consists of IDRs, a technique to control LLPS optically has been developed (Shin et al., 2017). This technique, called optoDroplet, is superior in that it can control LLPS with a high spatiotemporal resolution. A natural photosensor protein cryptochrome 2 from *Arabidopsis thaliana* shows the clustering in the cell (Yu et al., 2009), which might be similar phenomenon to LLPS. The TG method is surely applicable to such light-induced assembly systems.

## Studies on Non-Photoreactive Proteins

### Combining the TG Method With a Stopped Flow Apparatus

Although the TG technique is powerful, the target reaction must be initiated by light in principle. Hence, the target reactions have been limited to photochemical reactions. In order to detect time-resolved intra- and intermolecular reactions of non-photoreactive proteins, it is necessary to apply an external perturbation instantaneously to initiate a reaction. A high-speed mixing using a stopped-flow (SF) apparatus, which is designed to stop the flow of a solution immediately after high-speed mixing of two or more arbitrary liquids is appropriate for this purpose (Gomez-Hens and Perez-Bendito, 1991; Bleul et al., 2011; Zheng et al., 2015). In general, the reaction initiated by mixing is observed as a change in light absorption or fluorescence. Here, a TG technique using the SF system to detect the reaction initiated by the mixing (SF-TG method) is described.

Even if the biomolecular reaction is initiated by the high-speed mixing, changes in the refractive index of the solution must be induced by the photoexcitation to observe the TG signal. If the molecule is photoreactive, such as a photosensor protein, it is straightforward to obtain the TG signal, but if not, it is necessary to label the protein with a photoreactive molecule. For example, when a protein is labeled with a photochromic probe molecule, the light-induced change in the absorption spectrum creates the spatial modulation of the refractive index, which acts as a grating to produce the TG signal (Eitoku and Terazima, 2008; Takaramoto et al., 2020a). The diffracted light intensity then decays due to the diffusion. By analyzing the decay rate, the diffusion coefficient can be determined with a high sensitivity and in a short time (Figure 2B).

**FIGURE 8:**
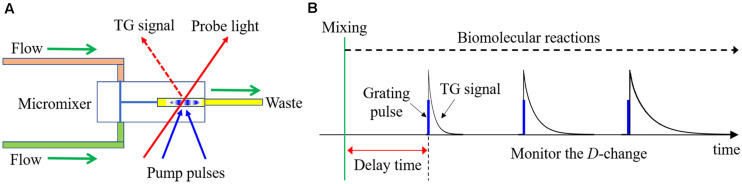
**(A)** Outline drawing of the SF-TG system. **(B)** Schematic illustration of the SF-TG method. The reaction is initiated by the rapid mixing, which is detected as the change of diffusion coefficient (decay rate of TG signal).

In the SF-TG method, a reaction is started by a mixing of two solutions, and excitation light for the TG measurement is introduced at various delay times to monitor the *D*-change during reactions (Figures 8A,B). From the *D* values, the higher-order structure of the transient state can be estimated and the rate of the *D*-change gives the kinetic information of the reaction. In the case of reversible reactions, the relaxation time after the equilibrium jump can be obtained. If the equilibrium constants are known from other measurements, the rate constants for the forward and reverse reactions can be determined separately from the relaxation time and an appropriate reaction model. In the case of intermolecular reactions, the rate constants for the association and dissociation reactions can also be determined individually from the concentration dependence on the relaxation time.

Practically, there had been two problems in the SF-TG method for application to protein reactions by commercially available SF systems; i.e., sample volume and time resolution. Usually, preparation of a large volume of protein solution for the SF system is difficult. Furthermore, the turbulent flow should be ended quickly in the mixing chamber before the diffusion measurement, and it was difficult using the traditional SF systems. To solve these problems, a micro-stopped-flow (μ-SF) system has been developed (Nakasone et al., 2019). In this system, the two sample solutions are quickly introduced into an observation cell using compressed air. The total volume of the mixer and the observation cell was about 3 μL, by which sample consumption can be significantly reduced. Furthermore, the small volume is appropriate to stop the turbulence quickly. The deadtime for the diffusion measurement was determined to be 100 ms. If this system is used for absorption or fluorescence measurements, which is not disturbed by the turbulent flow, the deadtime was estimated to be 400 μs. Here, as an example of the application of the SF-TG method, the conformational change of α-Synuclein upon binding of SDS micelle is presented.

### Folding Reaction of α-Synuclein Upon Binding of SDS Micelle

αSyn is a relatively small protein consisting of 140 residues and it localizes at the presynaptic terminals of neurons (Maroteaux et al., 1988; Weinreb et al., 1996; Breydo et al., 2012). The recombinant protein exists as a monomer with a disordered structure (an intrinsically disordered protein) (Fauvet et al., 2012). αSyn has attracting many researchers, since it is related with Parkinson’s disease (Polymeropoulos et al., 1997; Krüger et al., 1998). Recombinant human αSyn has been shown to form toxic oligomers and amyloid fibrils (Lashuel et al., 2013; Bengoa-Vergniory et al., 2017), which bind to lipid bilayers or vesicles (Pfefferkorn et al., 2012; Iyer and Claessens, 2019). Though αSyn forms a random coil in aqueous solution, particularly the N-terminal region forms helical structures upon association with negatively charged vesicles (Jao et al., 2004; Bekshe Lokappa and Ulmer, 2011). The conformational change is also induced by interaction with sodium dodecyl sulfate (SDS), which has been widely used in biophysical studies to mimic the membrane interaction of αSyn. With increasing the concentration of SDS ([SDS]), αSyn forms a complex with SDS and its N-terminal structure is changed from the random coil to an extended helix (Ferreon and Deniz, 2007). At higher [SDS], αSyn forms a bent (horseshoe) helix by the interaction with the micelles (Ulmer et al., 2005). Although previous studies investigated the structure of αSyn in the complex with SDS, the target has been limited to the static structures under equilibrium conditions. To clarify the dynamics of the binding and folding process, the intra- and intermolecular reaction of αSyn upon mixing with SDS micelle was studied by the SF-TG method (Takaramoto et al., 2020b).

**FIGURE 9:**
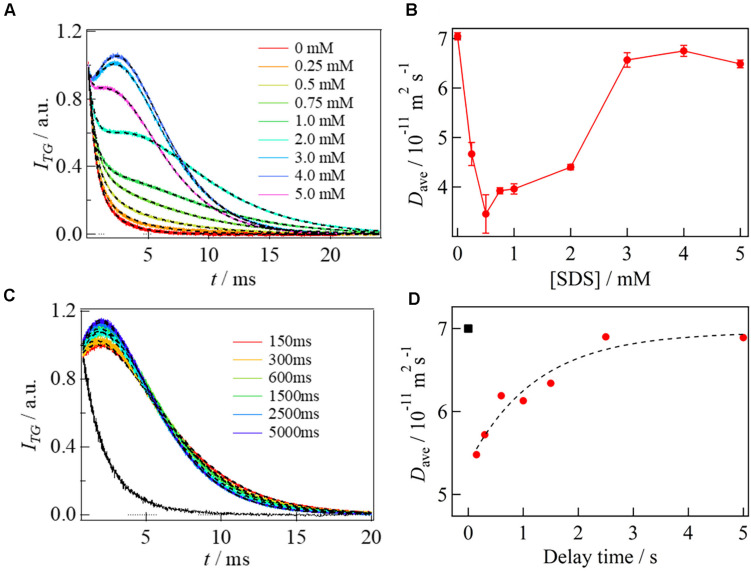
**(A)** [SDS] dependence of the diffusion signals of αSyn. The SDS concentrations are represented in the figure. The fitted curves by Eq. 2 are shown by the broken lines. **(B)** Plot of *D*_ave_ of αSyn against [SDS]. **(C)** The delay time dependence of the diffusion signal after mixing αSyn with SDS solutions (delay times are represented in the figure). A black solid line shows the signal obtained by mixing αSyn with PBS buffer in the absence of SDS at delay time of 5000 ms. The best fitted curves by Eq. 2 are shown by the broken lines. **(D)** Plot of *D*_ave_ against the delay time (filled circles). The broken line shows a fitted curve by a single exponential function. The calculated *D* of the unfolded state is shown by a black square at *t* = 0. All figures are adapted from Takaramoto et al. (2020b) with slight modifications.

For the TG measurement, the C-terminal end of αSyn was labeled with a small photochromic molecule of spiropyran (Klajn, 2014; Takaramoto et al., 2020a, b). Upon photoexcitation at 308 nm, the colorless ring-closed spiropyran (SP) form is converted to the violet-colored ring-opened merocyanine (MC) form, which causes a strong TG signal. Figure 9A shows the diffusion signal of αSyn labeled by the spiropyran and its dependence on [SDS]. The signal showed a monotonous decay without SDS, while a rise-decay profile appeared and gradually increased with increasing [SDS]. The rise-decay curve implies that *D*_P_ and *D*_R_ are different. This indicates that the transition from SP to MC forms of spiropyran affects the protein structure and leads to a slight change in *D*. The signals were analyzed based on Eq. 2 and averaged value of *D*_P_ and *D*_R_ [*D*_ave_ = (*D*_P_ + *D*_R_)/2] was used as the diffusion coefficient of αSyn under various SDS concentrations (Figure 9B). *D*_ave_ drastically decreased in the [SDS] region of 1–2 mM, and increased from 2 to 3 mM. The *D* change region agrees with the change of the secondary structure monitored by the circular dichroism (CD) measurement. The CD intensity representing the contents of the a-helix increased with increasing [SDS] by 1–2 mM and slightly decreased from 2 to 3 mM. Therefore, the initial decrease and the subsequent increase of *D*_ave_ are attributed to the extended helix formation and to the bent-horseshoe helix formation, respectively.

To investigate the kinetics of this protein reaction, the SF-TG measurements were used. Upon mixing of two solutions of the SP labeled αSyn and 6 mM SDS solution, the concentration of SDS is decreased to 3 mM rapidly, which is still above the critical micelle concentration (1.3 mM). Rapidly after this concentration jump, a profile significantly changed compared with that before mixing. It showed a rise-decay signal, and the profile gradually changes around 150–5000 ms (Figure 9C). The diffusion time of the TG measurement (∼10 ms) was sufficiently short compared with the delay time. Therefore, the signal was analyzed only by the diffusion process without any effect of reaction kinetics. The *D*_ave_ were plotted against the delay time and the time dependence was analyzed by a single exponential function (Figure 9D). The *D* values of the first intermediate state within the dead time of the SF system (I-state) and the final state were determined to be 5.5 × 10^–11^ m^2^ s^–1^ to 6.9 × 10^–11^ m^2^ s^–1^, and a rate constant of the reaction was determined to be 0.8 s^–1^. *D* decreases once upon the forming of I-state, and it increased to the final state of the complex.

**FIGURE 10:**
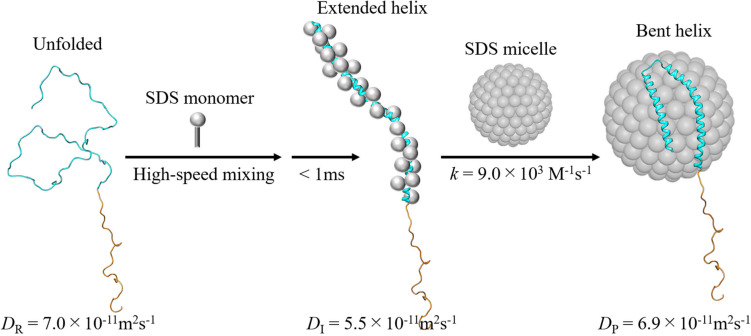
Proposed reaction scheme of the intra- and intermolecular reactions upon mixing of αSyn with the SDS. The α-helix formation is completed by binding to the SDS monomers, followed by association with the micelle to form the bent structure (Takaramoto et al., 2020b). A solution NMR structure of micelle-bound αSyn (Protein Data Bank Entry 1XQ8) is used as the structure of final product. For reactant and intermediate, artificial model structures are used.

The stopped-flow-kinetic measurements of CD and intramolecular FRET were also used for further detecting the conformation change. These measurements showed that the I-state forms the extended helical structure, while the final product forms the bent helix. Usually, *D*s of folded proteins are larger than those of unfolded proteins. The *D* decrease in the I-state may represent that a large aggregate is formed via intermolecular interaction with many SDS monomers. The increase in *D* upon formation of the final product may be explained by a compact (bent helix) form on the micelle. If αSyn forms aggregate with SDS monomers in the I-state, the complex formation with the micelle requires the release of these monomers. It has also been reported that the interaction of αSyn with the micelle leads to a shape deformation of the micelle to a prolate ellipsoidal structure. These factors may be origins of the observed slow rate of the transition from the intermediate to the final state. The proposed reaction scheme is shown in Figure 10.

It has been reported that misfolded αSyn is a major component in the Lewy bodies, which are related to Parkinson’s disease (Breydo et al., 2012). Recently, it has been shown that LLPS of αSyn precedes its aggregation. Factors known to promote αSyn aggregation, such as low pH, phosphomimetic substitution and familial Parkinson’s disease mutations, also promote αSyn LLPS and its subsequent maturation (Ray et al., 2020). It has also been reported that the maturation process towards the amyloid state is delayed in the presence of vesicles *in vitro* (Hardenberg et al., 2021). Therefore, the intermolecular interaction with the SDS micelle and folding of αSyn should be related to the suppression of the maturation process. Since LLPS of αSyn can be easily promoted by an addition of polyethylene glycol (PEG)-8000 (Ray et al., 2020; Hardenberg et al., 2021), it would be interesting to detect the conformational change and assembly process for LLPS by the SF-TG method. By monitoring this reaction in the presence of vesicles or SDS-micelles, the suppression mechanisms of LLPS will be possibly clarified based on the kinetic information.

## Future Perspective

The time-resolved diffusion technique is a promising tool for studies on biomolecular reactions including LLPS, because *D* is sensitive to conformation changes, intermolecular interactions, and complex formations. It has been difficult to detect the dynamics of these properties using other time-resolved techniques. Furthermore, using the SF-TG method, the LLPS formation process can be initiated by a mixing with partner molecules, pH jump, change of salt concentration, etc. Although IDRs lack stable tertiary structure, they may undergo disorder-to-order transitions upon binding to partners. From the kinetic analyses, it would be able to distinguish whether the intermolecular contacts are necessary before forming a folded structure (induced-fit mechanism) or the folding precedes the formation of intermolecular contacts (conformational selection mechanisms). The information is important for understanding of molecular recognition mechanisms during the LLPS.

Fluctuations are an intrinsic property of biomolecules and are essential for achieving high reactivity of biosystems. Although the diffusion measurement was mainly described in this review, the TG method is also able to determine thermodynamic properties closely related to fluctuations, such as compressibility and thermal expansion coefficient, for reaction intermediates (Terazima, 2002, 2004; Eitoku et al., 2007; Kuroi et al., 2014, 2016; Nakajima et al., 2016; Iwata et al., 2018). It has been shown that the transient increase in fluctuation is a driving force for the subsequent reaction, and that the reactivity and enzyme activity decrease under the conditions where the fluctuation is suppressed. These facts suggest that the fluctuation is an important property for reaction and function of biomolecules. IDRs have high flexibilities and always fluctuate in solution and in living cells, which should be a key factor to achieve the fluidity and reversibility of LLPS, and the loss or suppression of flexibility may be related to the amyloid formation. We hope that the detection of conformational fluctuations and molecular assembly/disassembly processes by the TG method will help us to achieve hierarchical and kinetic understanding of LLPS at the molecular level.

## Author Contributions

YN conceptualized the review and wrote it with MT. Both authors contributed to the article and approved the submitted version.

## Conflict of Interest

The authors declare that the research was conducted in the absence of any commercial or financial relationships that could be construed as a potential conflict of interest.
